# Access to healthcare services during the COVID-19 pandemic: a cross-sectional analysis of income and user-access across 16 economically diverse countries

**DOI:** 10.1186/s12889-024-20147-y

**Published:** 2024-10-01

**Authors:** Zachary D. V. Abel, Laurence S. J. Roope, Raymond Duch, Philip M. Clarke

**Affiliations:** 1 Health Economics Research Centre, Nuffield Department of Population Health, University of Oxford, Oxford, OX3 7LF UK; 2 National Institute for Health Research Oxford Biomedical Research Centre, John Radcliffe Hospital, Oxford, OX3 9DU UK; 3 Nuffield College, University of Oxford, Oxford, OX1 1NF UK; 4 Centre for Health Policy, Melbourne School of Population and Global Health, University of Melbourne, Melbourne, VIC 3010 Australia

**Keywords:** COVID-19, Socioeconomic inequality in health, Health access

## Abstract

**Background:**

National health systems have different strengths and resilience levels. During the COVID-19 pandemic, resources often had to be reallocated and this impacted the availability of healthcare services in many countries. To date there have been few quantitative contemporary studies of inequalities in access to healthcare within and between countries.

In this study, we aim to compare inequality within and between 16 economically diverse countries.

**Methods:**

Online surveys were conducted on 22 150 adults in 16 countries across six continents in 2022. Quota sampling and post-stratification weighting was used to obtain an age, gender, geographically, and educationally representative sample. The study assesses the differences in challenges in access to healthcare during the pandemic (for GP, surgical/clinical and digital GP services) using country-specific expanded health-needs-adjusted Erreygers’ concentration indices and compares these values between countries using a Spearman’s rank correlation coefficient.

**Results:**

Results show wide variation in income-related challenges in access within countries for different types of care. For example, Erreygers’ concentration index for digital services in Colombia exhibited highly regressive inequality at 0·17, compared to Japan with an index of -0·15. Inequalities between countries were also evident, with Spearman rank coefficients of -0·69 and -0·65 (*p*-values of 0·003 and 0·006) for digital and surgical access, indicating that lower income countries had greater inequality in healthcare access challenges.

**Conclusion:**

During the pandemic, inequalities in challenges to accessing healthcare were greatest in low and middle-income countries. Digital technologies offer a reasonable means to address some of this inequality if adequate support is provided and accessible digital infrastructure exists.

**Supplementary Information:**

The online version contains supplementary material available at 10.1186/s12889-024-20147-y.

## Introduction

The onset of the COVID-19 pandemic saw many health systems overwhelmed in ways that few would have expected. At its extremes, when care was required, certain systems, e.g. in Italy and India among others, were unable to provide care adequately due to the number of COVID patients requiring attention [1, 2]. The concept of limited resources in healthcare is familiar to many health systems and practitioners who work in these systems. In these systems, it is often necessary to take decisions that prioritise resources, which can result in individuals receiving, postponing, or being refused care – as in triage environments. While changes in overall utilisation of healthcare have been documented during the pandemic [3], how these changes translated to challenges for individuals in accessing care at different levels of income has not been studied. It is possible that re-prioritisation associated with the pandemic may have led to changes in how equitably care is provided. Understanding the experiences of patients in diverse contexts is necessary to determine where inequality lies, and take the required steps to address inequality.

Health economics studies frequently employ the concentration index or related measures to quantify inequalities in access to different forms of healthcare, after adjusting for need [4]. However these studies are often limited in their use for global comparisons due to differences in methodology (e.g., different inequality measures), focus (types of access), and timing.

Healthcare access itself can be defined conceptually in several ways. In their 2013 study, Levesque et al. develop a framework that breaks healthcare access into 6 stages, beginning with health care needs, through perception of needs, seeking care, reaching care, utilisation, and outcomes or consequences [5]. The stage we seek to investigate is reaching care, conditional on having sought care. We investigate the extent to which people perceive difficulties in reaching care, and how this is associated with socioeconomic status. Our study includes individuals’ subjective perceptions on the difficulties in accessing care and, in this regard, is similar to works by Fjaer et al. and Cylus and Papanicolas [6, 7].

The literature on cross-country health access comparison is largely concentrated in specialised pathways, (e.g., maternal and child care, elderly care, insurance coverage) or within constrained geographic regions (e.g., Sub-Saharan Africa, OECD, Latin America) [8–12]. We conducted a literature search focused on the quantitative assessment of socioeconomic or income-related inequalities in access to health services, limiting the search to results from 2020 onwards to maintain relevance to the COVID-19 pandemic time-period. After an extensive screening and review process, we identified several papers that focused explicitly on barriers to access and unmet need and were published in the pandemic time-period. Unmet need has been defined as needing care and not receiving care, either as a self-determined requirement (e.g. in works by Gordon et al., Houghton et al., and Wu et al., [13–15]) or if referred to care by a doctor (e.g. in Zhuoga et al.’s work) [16]. Alamneh et al. define a challenge to access as being a situation where individuals faced financial constraints, long distances to facilities, did not receive permission to consult a health practitioner, or did not want to attend an appointment by themselves [11]. Alamneh et al. investigate barriers in accessing maternal care in Sub-Saharan Africa [11]. Ghana, Uganda, and South Africa are included in their study, alongside 30 other African countries. They find pro-rich inequality in accessing maternal care, though estimates at the country level were not available. Houghton et al. conduct an analysis on access barriers in four Latin American countries, including Colombia. Lower-income respondents faced increased barriers relative to wealthier respondents, with significant results observed from 2010–2016 using the slope index of inequality. Wu et al. investigate unmet need for inpatient and outpatient care in China from 2011–2015, while Zhuoga et al. investigate unmet need for hospitalization (inpatient) in Tibet [14, 16]. Wu et al. find inequality favouring the rich for both inpatient and outpatient unmet need due to financial constraints, but find that the poor were less affected than the rich by non-financial constraints. Their work implies that financial barriers reduce care-seeking more than non-financial barriers for low-income earners [14]. Zhuoga et al. find pro-rich inequality in unmet need, implying that the poor had disproportionately more unmet need, though the result was not statistically significant. The pro-rich inequality decreased marginally over the study period (2013–2018). In a 2012 study, Gordon et al. find pro-rich inequality in unmet need in South Africa. Similarly, postponement of healthcare was also inequitably concentrated amongst less-wealthy households [13]. A further study by Nontarak et al. investigates modes of medication delivery during the pandemic, and found that traditional services for non-communicable disease patients exhibited pro-rich inequalities. However, new modes of medication access such as primary care facility collection, postal delivery, and delivery by volunteers exhibited pro-poor inequalities [17].

Current COVID-related cross-country research focuses predominantly on health outcomes and utilisation rather than challenges in access [18–20]. None of the papers we identified in the literature search that focused on unmet need used data from the COVID pandemic period. This quantitative multi-country study aims to address this gap in the literature. We apply the Erreygers concentration index to assess inequality in the perceived challenges faced in accessing needed care during the COVID-19 pandemic [21]. The results of this investigation display how healthcare users experienced their country’s healthcare system during a period of the COVID-19 pandemic, and can provide insight into future health shocks. We investigate healthcare challenges after seeking care and before reaching care, as per Levesque et al.’s conceptualisation of access [5], across three types of care: in-person GP appointments; telephone or internet (digital) appointments; and surgical or clinical admissions. Individuals rated the ease with which they were able to obtain appointments (if required) in each type of care, and these responses were analysed with respect to household income to determine inequality levels. The subjective nature of this question separates this study from previous work. Our study focuses on challenges accessing care for those actively seeking care, rather than on general and persistent barriers to care, which is the focus of many studies. After estimating within-country inequality in each of the three investigated types of care, we analyse patterns in between country inequalities with respect to median national household income, using Spearman’s correlation tests.

## Methods

### Study design, setting and populations

This investigation was conducted using data collected in the second wave of the COVID-19 Vaccine Preference and Opinion Survey (CANDOUR) study. CANDOUR is an online, longitudinal, multi-country survey for adults aged 18 and over. All participants provided informed written consent before beginning the survey. The wave 2 survey was developed for this and other studies as a follow-on to CANDOUR wave 1. The second wave of the CANDOUR study was expanded from vaccine preferences to investigate public attitudes related to the COVID-19 pandemic more broadly, and included questions that we developed specifically to assess public attitudes on difficulties in accessing care during the pandemic.

**Fig. 1:**
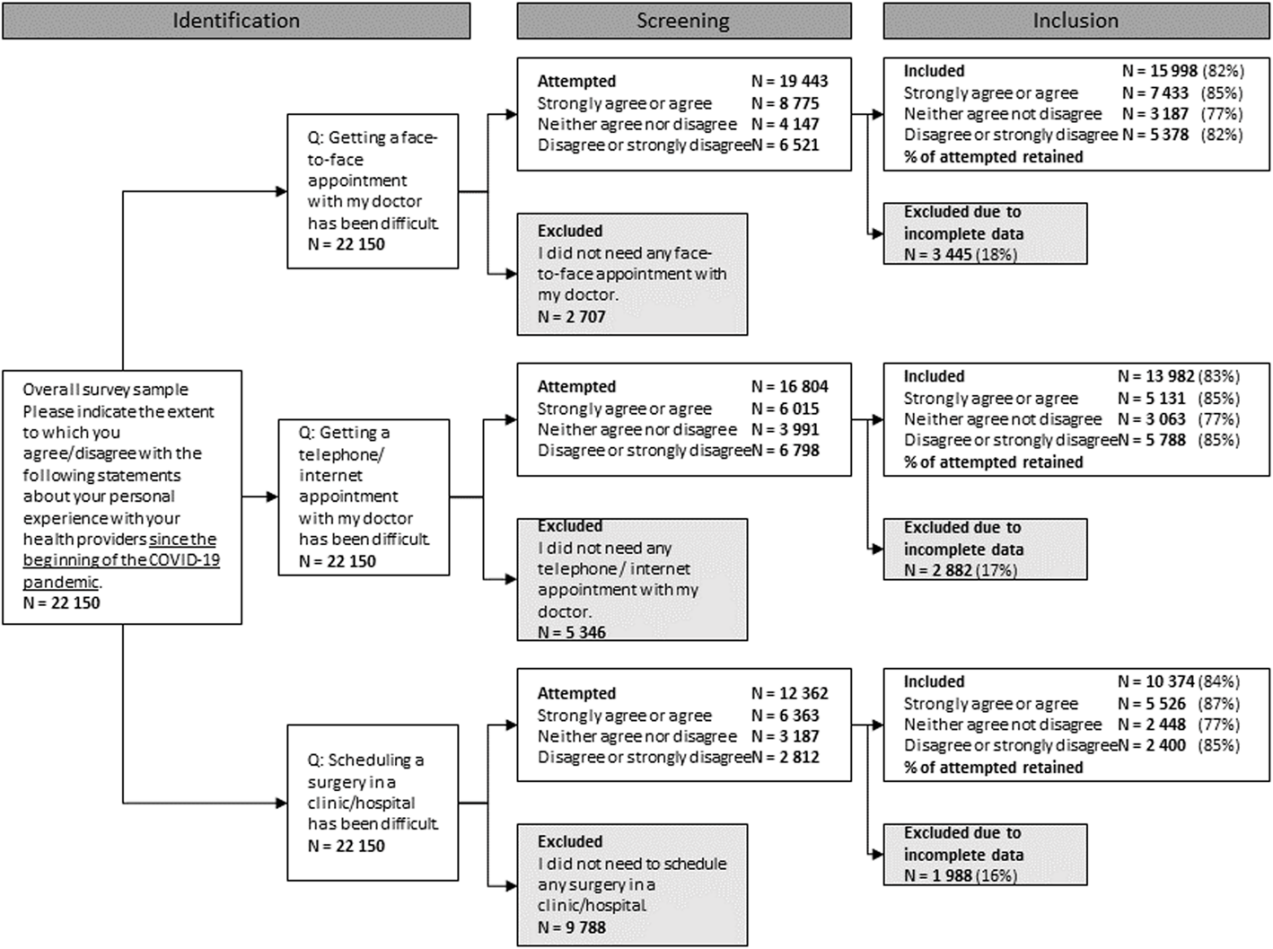
Individual respondent criteria for inclusion in analysis flowchart

The wave 2 survey was conducted in 16 economically diverse countries using anonymous online surveys from March to November 2022 [22–25]. The relevant questions from the wave 2 survey instrument are included as a Supplementary file. 22 150 responses were collected, averaging 1385 per country, ranging from 1266 to 1907. The CANDOUR study used quota sampling to ensure representative samples in terms of gender, age, education, and geography in each country. Where imbalances remained, post-stratification weighting was adopted. Analysis was restricted to individuals who attempted to engage with each type of care, and with complete data for the demographic and health standardization variables. This limited respondents for each of the challenge types to 15 998 for face-to-face GP challenges, 13 982 for digital appointments, and 10 374 for surgical/clinical appointments. The selection methodology and exclusion criteria are presented in Fig. 1*.*

### Data collection

We asked respondents to rate their experience of getting an appointment in each of three types of care since the beginning of the COVID-19 pandemic, to assess challenges in accessing care. Respondents were asked the extent to which they agreed or disagreed with a statement (e.g., ‘getting a face-to-face appointment has been difficult’) from ‘strongly agree’ to ‘strongly disagree’, with five inclusive ordinal steps, and an option if they did not need care. Responses of ‘strongly agree’ and ‘agree’ were classified as having faced challenges for each type of care. Participants who did not attempt to make appointments were excluded from the analysis of the relevant type of care. Annual household income was self-reported in income bands. This metric was equivalised using the square-root of household size [26], and converted to purchasing power parity values to allow comparison between countries. All other individual variables used were self-reported in the survey, while a number of sources were used for external aggregate data.

### Statistical analysis

The primary purpose of the analysis was to determine the relationship between income and challenges in health access, within and across countries. Concentration indices were computed for each health-needs-adjusted challenge variable, for each country, using annual equivalised household income as the ranking variable. The challenge variables were indirectly standardized for health needs using non-linear regression methods, within each country to maintain specificity for the purpose of cross-country comparison. Country specific standardization was conducted accounting for gender, age, current health level, number of chronic conditions, COVID risk, and willingness to risk health. We include the COVID risk and willingness to risk health variables to account for individual likelihood of seeking appointments, as self-reported attitudes to risk have been shown to play a role in health related behaviour and are likely to play a role here [27, 28]. For instance, individuals less willing to risk health and at higher perceived risk of contracting COVID may be more likely to seek medical appointments. We further controlled for education level, marital status, political ideology, labour force participation, and household size within each country. We use an expanded set of variables for standardization to account for differences in how respondents may perceive challenges in accessing care [4]. We provide our rationale for inclusion of variables in the expanded standardization set (Supplementary Tables S7 and S8), and use a restricted standardization of age, gender, and health status as a robustness and sensitivity analysis, both of which are reported in Supplementary materials (Figures S2-S4; Table S6). The Erreygers adjusted concentration index was used given its desirable properties for binary variables, its scale invariance (a necessary characteristic to ensure equivalent comparisons across countries, which may have different means), and quasi-absoluteness [21]. The index is calculated as follows:

$${E}_{j}({\text{h}}_{\text{j}}) = \frac{8}{{{n}_{j}}^{2} ({b}_{hj}- {a}_{hj})}\sum_{j=1}^{j}\sum_{i=1}^{n}{z}_{ij}{h}_{ij}$$

where $i$ is the individual respondent; $j$ is the country; ${h}_{ij}$ is the measure of the standardized health variable (challenge status); ${b}_{hj}$ and ${a}_{hj}$ are the upper and lower bounds of ${h}_{ij}$ respectively; ${n}_{j}$ is the sample size and ${z}_{ij}$, a weighting variable of relative socioeconomic rank (equivalized household income in this analysis) [21]. We also employ the Wagstaff index in a sensitivity analysis and find no notable differences in our results.

We test for correlation between the computed indices within each type of care and median household income based on significant correlations in the regression to determine if there is a monotonic relationship across countries using the Spearman’s rank correlation coefficient. We include an analysis on medical practitioner density in place of median household income in the Supplementary materials, on the basis of the variable’s significance in the regression.

All statistical analyses were conducted using Stata 17.0 (StataCorp LP; College Station, TX).

### Patient and public involvement

No patients (respondents) or members of the public were involved in the study design or data analysis.

## Results

### Sample characteristics

The full wave 1 sample descriptive statistics are displayed in Table 1, while sub-group sample descriptive statistics are available in the supplementary materials (Tables S1-S3). All selected variables were reported as mean values apart from age, household income, education level, and political ideology, which were reported as medians. We report standard deviations alongside means and interquartile ranges alongside medians. Where variables are binary, the stated values represent the percentage of individuals in a country with the stated attribute. The overall study sample is 50% female. The median respondent has completed secondary education and is 39 years old. 70% of the sample is economically active in the labour force. Average self-reported health on a scale of 0–100 (where 100 is perfect health) is 68.9%. The average respondent had 0.6 chronic conditions, with an average willingness to risk health of 3.5/10. The samples within individual countries in Table 1 varied. Comparisons between countries and subgroups are left to the interested reader using Tables 1 and S1-S3. Respondents that attempted to seek any care (*n = *16,367), and thus were included in our study, are on average lower income earners, in worse self-perceived health, younger, more likely to be employed, slightly better educated, more likely to be male, and more willing to take risks with their health than those who did not attempt to seek care (*n = *5,783). Results of statistical tests between the sub-samples, and the observed differences are available in Table S4.

**Table 1:** Overall sample descriptive statistics

	**Median Age (years)**	**Female (%)**	**Median Education level**^**a**^	**Med. Income (PPP$ ‘000 s)**	**Labour force participation (%)**	**Self-reported health (%)**	**No. Chronic conditions (n)**	**Willingness to risk health**^**b**^
Overall	39(27–55)	50(50)	3(3–4)	14·7(4–32)	70·0(46)	68·9(27)	0·60(0·9)	3.5(2·9)
Australia	41(28–56)	59(49)	3(3–4)	28·6(16–57)	67·8(46)	64·7(25)	0·74(0·9)	4·1(2·9)
Brazil	33(26–54)	54·9(49)	3(1–3)	5·3(2–9)	53·7(49)	66·8(26)	0·62(0·9)	3·2(3·0)
Canada	30(23–55)	65·8(47)	4(3–4)	35·7(14–50)	74·4(43)	70·8(25)	0·59(0·8)	3·4(2·4)
Chile	32(25–43)	57·8(49)	3(3–4)	12·2(8–20)	68·8(46)	72·6(20)	0·72(0·7)	3·2(2·6)
China	43(28–53)	49·2(50)	3(3–3)	20·5(13–28)	73·1(44)	78·3(21)	0·35(0·6)	3·3(3·1)
Colombia	48(31–60)	49·7(50)	3(2–3)	8·9(4–14)	65·5(47)	59·7(35)	0·58(0·8)	3·4(2·6)
France	49(35–61)	58·7(49)	3(3–4)	29·2(15–46)	54·1(49)	68·9(25)	0·53(0·7)	3·5(2·8)
Ghana	30(26–34)	19·8(39)	4(3–4)	0·9(0·5–5)	75·7(42)	84·6(17)	0·17(0·4)	3·3(3·2)
India	31(25–40)	45(49)	4(2–4)	3·5(2–10)	87(33)	49·5(35)	0·75(1·0)	5·5(2·8)
Italy	52(40–62)	57·6(49)	3(3–3)	24·1(16–37)	56·7(49)	69·8(22)	0·65(0·8)	2·9(2·8)
Japan	55(36–65)	52·2(49)	3(3–4)	31(12–45)	77·2(41)	69·1(23)	0·66(0·9)	3·9(2·5)
South Africa	36(27–47)	46·8(49)	3(2–3)	8·9(0·1–24)	79·4(40)	69·2(27)	0·77(1·2)	2·6(2·4)
Spain	44(33–61)	48·4(49)	4(3–4)	30·5(22–54)	47·3(49)	70·7(28)	0·48(0·7)	2·8(2·7)
Uganda	28(25–36)	25(43)	3(3–3)	0·2(0·06–0·5)	91·3(28)	73·8(22)	0·34(0·7)	3·1(2·6)
UK	42(32–51)	52·9(49)	3(3–4)	23·5(11–41)	76·8(42)	64·5(23)	0·68(0·9)	4·2(2·9)
US	44(28–59)	52(49)	3(3–4)	35(18–51)	70·4(45)	69·5(23)	1·02(1·0)	3·4(2·8)

Note: Figures are presented as mean(SD) and median(IQR)

^a^1 = less than primary completed; 2 = primary school completed; 3 = secondary school completed; 4 = university completed

^b^Respondents were asked how willing they were to risk their health from 0 to 10

### Inequality of access

**Fig. 2:**
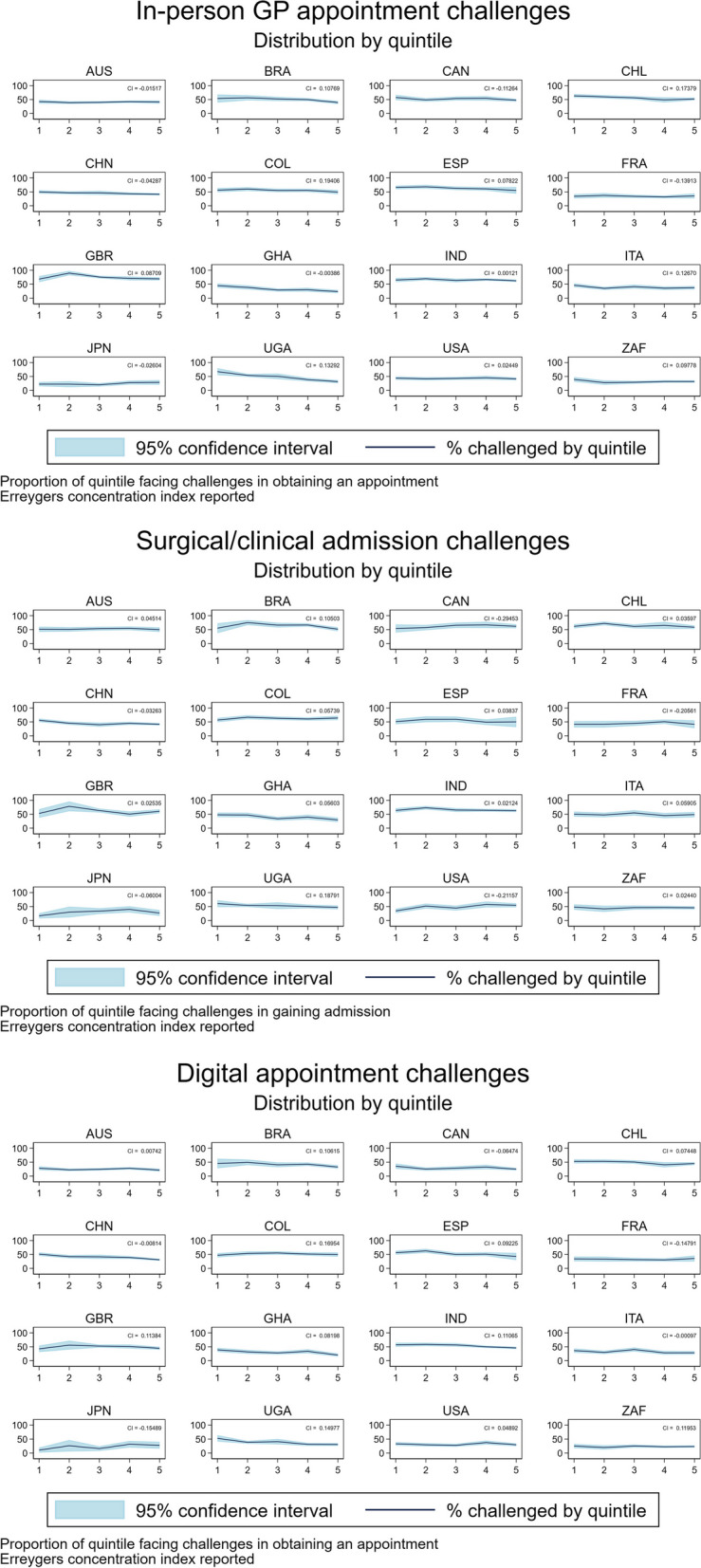
Graphical representation challenge statistics by type of care, quintile, and country

Healthcare challenges to access were investigated across income quintiles (Q1 = lowest income quintile; Q5 = highest income quintile) within each country, conditional on attempting to interact with the respective type of care. We observe ranges in the proportion of populations challenged while seeking care from 21% (Japan Q3) to 89% (United Kingdom Q2); 17% (Japan Q1) to 78% (United Kingdom Q2); and 10% (Japan Q1) to 63% (Spain Q2) in the in-person GP, surgical or clinical, and digital care types respectively. These results, shown graphically in Fig. 2, present the gradient of inequality within each country across each of the types of care. Uganda and Ghana show increasing ease of access with income across each type of care, evidenced by the downward sloping “challenged” lines. Concentration indices were calculated, and are estimated on each of the panels in Fig. 2, providing firm numerical estimates of within country inequality.

**Fig. 3:**
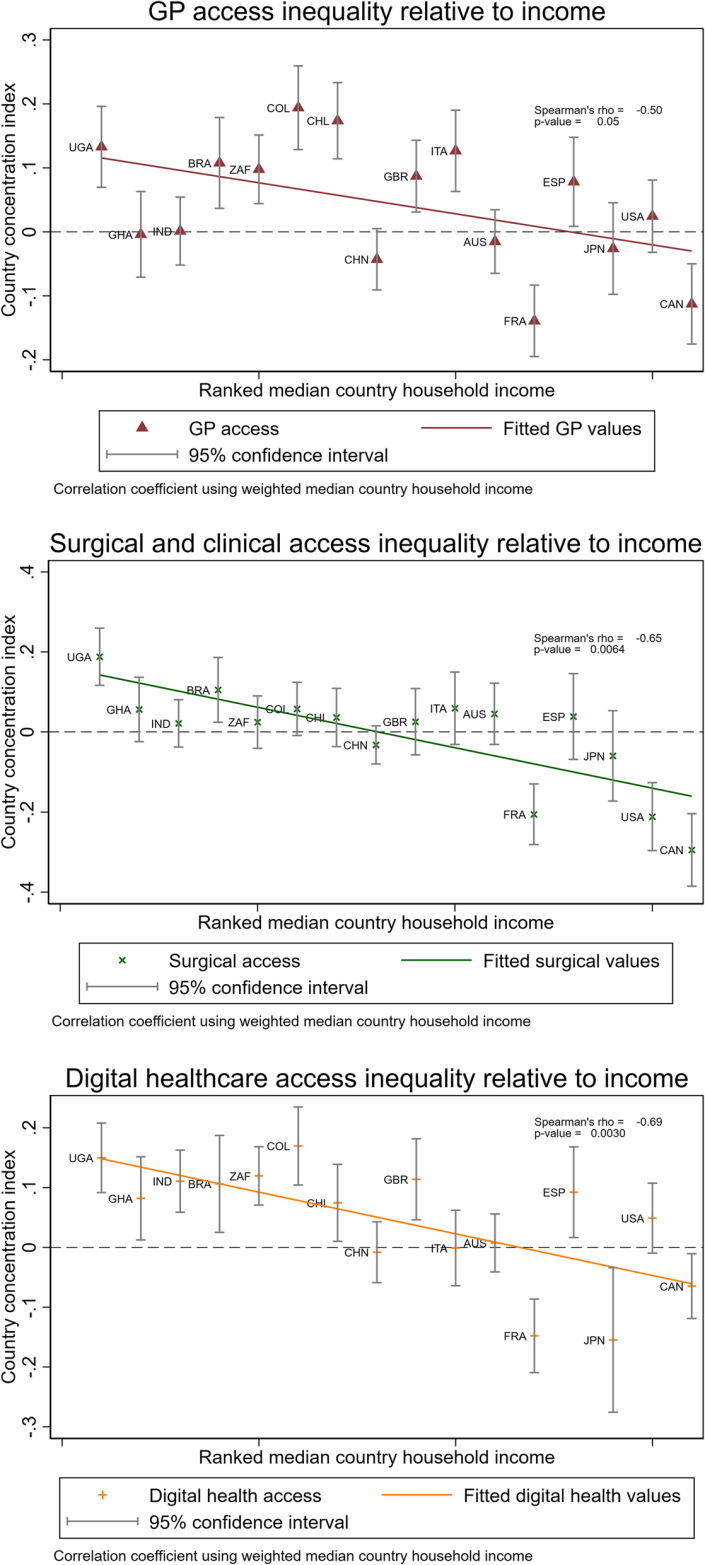
Challenge inequality by type of care and country-ranked median income levels

The results of the computed concentration indices across countries are reported graphically in Fig. 3 against median household income, as median income is shown to be correlated with inequality in access challenges across the three types of care in a country level regression. The regression results are available in Table S5. Within country inequality is presented in Fig. 3, with the country’s concentration index plotted on the chart as a point on the y-axis, with a 95% confidence interval. Results using the restricted standardization are reported in Figures S2-S4. Correlations with income are robust to standardization, apart from the in-person GP results (Figure S2). We observe ranges in the inequality indices from 0·19 (Colombia) to -0·14 (France); 0·19 (Uganda) to -0·29 (Canada); and 0·17 (Uganda) to -0·15 (Japan) in the in-person GP, surgical or clinical, and digital care types respectively.

In Fig. 3*,* between country inequality and within-country inequality are shown relative to median household income. Between-country inequality is shown by the trend-line and the correlation measure between the inequality indices and median national household income. Results are negatively sloped in each of the types of care investigated. Median income has the largest observed correlation in the digital care-type, with a calculated Spearman’s correlation coefficient of -0·69 and p-value of 0·003. The surgical/clinical type of care follows closely with a correlation of -0·65 and p-value of 0·006. Results in the GP channel are weaker. The negative monotonic relationship between median income and inequality is observed across each of the variables investigated, effectively implying that lower median country income is associated with higher inequality in reported challenges to accessing care during the COVID-19 pandemic.

## Discussion

This study investigated the relationship between income levels and inequality in accessing health services in three different care pathways across 16 socioeconomically diverse countries. In around half the countries studied, we observe significant inequalities in access to care based on the difficulties in accessing care reported by respondents. In terms of understanding global patterns of inequality between countries, a regression analysis indicated that country level median income was most strongly correlated with inequalities in access. For example, we observe a correlation of -0·69 between our measure of national median household income and inequality in challenges to access to internet or telephone (digital) appointments, and a correlation of -0·65 in challenges in obtaining surgical or clinical admissions, suggesting that citizens in lower- and middle-income health systems faced greater inequality in accessing care during the COVID-19 pandemic (the World Bank country income classification is available in Table S9).

While there is a significant literature on inequality for particular types of care within individual countries and regions [8–12, 29, 30], globally representative quantitative analyses during and post the COVID-era are rare. Though there is a growing body of research linking inequality in health outcomes during the pandemic, investigations on access remain limited. The strength of this study lies in the ability to compare inequality across 16 countries, accounting for roughly half of the world’s population, for challenges in three different types of care (in-person GP care, surgical or clinical care, and digital care). The three types of care investigated cover a significant portion of patients’ primary care contacts with the healthcare system, making them important markers of initial and subsequent access. The countries included in the analysis cover each of the World Bank country classifications by income, and are geographically diverse, covering each of the six continents (excluding Antarctica).

### In person-GP and surgical or clinical admissions inequalities in context

Research on access to healthcare during the COVID-19 pandemic is sporadic across countries and types of care. Several previous studies provide additional context or support to the results of our study. Research has found temporarily heightened inequalities in GP consultation utilisation in the UK [31], consistent with our estimates for inequality in challenges to in-person GP appointments during the pandemic. In the UK, the most deprived areas faced marginally larger reductions in hospital admissions than the least deprived areas, despite the higher incidence and mortality of COVID-19 among lower socioeconomic status groups [32–34]. These results and other previous work showing a socioeconomic gradient to accessing surgical care [35], support our surgical or clinical access inequality point estimate. Research has found that in Uganda, lack of financial resources was the primary reason cited for being unable to access medical treatment [36], consistent with our broadly unequal results found in each care type in the country. Research in South Africa suggests that observed worsening health inequalities during the pandemic were due to effects of lockdown, felt and borne disproportionately by the poor [37]. Our study supports the hypothesis that inequalities were borne more by the poor. Canada, Italy, Japan, and Spain each took measures to reach vulnerable groups during the pandemic, reducing challenges to access across the health system [38]. We reported pro-poor point-estimates in half of the instances concerning these four countries excluding the digital channel. Inequitable results broadly supporting our findings were observed in the south of Brazil for specialised health services, though we cannot perfectly match the types of care to our own [39]. A 2021 report addressing differences in health systems between the US and other high-income countries found Australia to provide the most equitable access to care, care process, and administrative efficiency [40]. The UK, France, and Canada followed, with the USA performing the worst and placing last. The 2021 study also investigated access to care in detail, combining metrics of affordability and timeliness. The UK placed first in access, driven by the affordability of the NHS system, followed by France, Australia, Canada, and the USA [40]. While it is difficult to draw direct comparison with our results due to the specific nature of the metrics used in each study, it is necessary to note the disparities. On average, across the three types of care we investigate, France and Canada are the most equitable, followed by the USA, Australia, and the UK. A possible explanation is that our study focuses on those who attempt to gain access to health systems and face challenges, but does not incorporate those who do not seek care initially due to affordability and other barriers, which is captured in the corresponding report [40]. A second potential reason for the observed disparities, aside from the metrics measured, are the methods employed; the 2021 report compares equity with only two income groups; above and below the median income [40], compared to our more granular measure using several income groups across the distribution.

### Contextualising the digital healthcare results

Digital health inequalities were not found in Japan in previous work during the pandemic, contrary to our own findings which showed pro-poor accessibility of digital services [41]. The surveys used in our study were conducted two years after the previous work in Japan, offering a possible timing explanation for the different results observed. Previous work has found that low-income ZIP codes were still less likely to seek care digitally compared to better-off counterparts in the USA during the pandemic [42], which is consistent with our finding in digital care in the USA. Temporarily increased inequalities in the use of tele-health services were found in the UK during the earlier stages of the pandemic [43], but this returned to equitable levels in subsequent months [31]. It is notable that several high-income countries took steps to improve digital accessibility during the pandemic to reach vulnerable citizens, including reimbursement and subsidisation of teleservices, practice guidelines, and removing requirements for in-person assessment for sick certificates [38]. Our study finds pro-poor digital challenge inequality estimates for three of the four countries that took measures to improve accessibility, suggesting that such policies may reduce health inequalities.

### Policy implications

Low income levels have been associated with higher incidence and mortality rates of COVID-19 [33, 34]. Many factors affect access to healthcare. In some cases, these factors are dependent on the definition of access, which varies in the literature [5]. Some factors that are frequently associated with socioeconomic status and are known to affect health access are affordability, proximity to health facilities, transport accessibility, education levels, and media exposure, amongst many others [11, 44] With higher incidence and reduced access to healthcare, as shown in this study, in certain countries, it follows that poor health outcomes will continue to be disproportionately distributed amongst the economically vulnerable [45]. This would result in a double burden of ill-health compared to wealthier counterparts, resulting in a “*syndemic pandemic*” [45]. This double burden will remain persistent unless policy can be implemented to expand and protect access to healthcare services and systems for the poor, addressing the inverse care law and ensuring horizontal equity [46]. Based on the monotonic relationships observed between national median income and inequality, lower-income countries in the study (on average) were less able to provide equitable care to citizens. Possible causes for this result include the existence of strong private systems accessible only to wealthier individuals in low-income countries (e..g, the case of South Africa), or the heightened presence of corruption associated with lower income countries, which may enable those who engage in corrupt activities to gain preferential access to health systems in times of need [47]. The development and implementation of digital health services offers a policy opportunity to address these inequities. These digital services (i.e., telephone and internet-based consultations) presently show large inequalities in lower-income countries, but may simultaneously offer future paths to mitigate inequality and inequity. Specifically, a digital offering can circumvent barriers in transport, logistics, and overall consultation duration to reduce required time off work. Digital distance-based services require only an internet or phone connection, access to which is increasing in developing countries [48].

Digital health services expanded rapidly during the pandemic to promote safety and protect healthcare workers [49, 50]. However, the role of digital health in the post-pandemic world is unclear and will continue to involve. Unlocking the potential inequity-mitigating effects of digital and distance services is likely to require significant support in implementation, particularly in resource-constrained systems. Lower-income countries in our study did not provide equitable digital services, while higher-income countries were more able to do so, and in some instances took specific interventions to improve digital access [38]. One such implementation support lever could involve the prioritisation of digital and distance services for rural (by distance from practice) patients, if not explicitly by income level.

As much of the world returns to pre-pandemic practices, the now-known possibilities of digital health will be included in the care offering available to patients. It is necessary that low-income countries and low-income communities are supported to improve access to digital health services to reduce inequity. Digital services offer an opportunity to reduce and reverse inequality if policy and initiatives surrounding rollout are well-managed and targeted, ensuring that marginal groups are not left behind [43, 51].

### Limitations

There are five limiting factors to consider in this study:

(1) Survival bias. The survey asks respondents if they had difficulty in obtaining an appointment or admission in three different care types. The analysis conducted assesses inequalities in challenges to obtaining access, but does not incorporate those who did not attempt to gain access to health systems but needed to, which may be due to traditional barriers to care, such as proximity, affordability, and time constraints [11, 44]. (2) The use of indices. It is important to note that the concentration index is a summary measure, and does not necessarily reflect intermediate information along the distribution. Examining the index value alongside a graphic aid is useful in understanding the overall distribution of inequality. Visuals may show switches between equality and inequality, whereas the index nets these values, such as in the case of separated public and private health systems accessed by different income groups [52]. (3) The cross-sectional nature of the study. This survey was conducted from March –November 2022, however this study lacks a prior counterfactual. As such, we can only infer that the observed inequalities were present during the period from the onset of the COVID-19 pandemic to the end of the study period. Without a prior counterfactual, we cannot ascertain the direction in which inequalities may have changed. We are similarly unable to ascertain whether the pandemic, or associated health system restructurings (e.g. increased digital consultations), had a causal impact, in either direction, on these inequalities and inequities. Further research to address the state of inequality following the pandemic is planned, employing subsequent waves of the CANDOUR survey. (4) Survey limitations. Respondents may misremember certain elements of their interactions with health systems over the ~ 2 year study period. Such recall errors would bias the results, though research has found that longer recall periods minimise bias [53]. The same research found greater measurement errors in lower-income groups. We recognise that the countries studied did not face the same infection or restriction profiles and, at the time they were surveyed, respondents may have found themselves in varying contexts. However, the ~ 2 year study period allows respondents to provide an overview of their experiences in accessing care in their country, from the beginning of the pandemic to the date of survey, which remained in the global health emergency phase of the COVID-19 pandemic as defined by the WHO [54, 55]. Respondents (or respondent groups) may have subjective views over what constitutes a challenge in accessing care, which may bias the results if systematically prevalent among groups. The subjectivity in this response is the underlying reason for inclusion of the expanded standardization set – aiming to collect information on respondents’ subjectivity based on a wider array of variables, and control for this. (5) Sample power at aggregate level. In testing between country differences we reduce the sample to 16 observations per type of care, rather than pooling the responses. This necessarily reduces the power of the test, which with a limited number of degrees of freedom may be unable to provide significant results despite indicated correlations.

These limitations notwithstanding, this study adds to the existing literature on inequality in access and challenges to care. The paper goes beyond a single-country analysis by exploring the relationships between three different types of care and income levels across 16 economically diverse countries. In doing so, it finds strong relationships between median income and healthcare accessibility challenges in digital healthcare services and surgical or clinical admissions. Given the rise of digital healthcare during the pandemic and its potential to alleviate inequality in health care access, the digital health services results in this paper should be given due consideration. Policymakers may be able to alter inequality gradients with intentional digital interventions, mirroring those countries that have successfully implemented equitable digital services.

## Supplementary Information

Supplementary Material 1.

## Data Availability

The datasets used and/or analysed during the current study are available from the corresponding author on reasonable request.
