# Supplementary material for: Access to healthcare services during the COVID-19 pandemic: a cross-sectional analysis of income and user-access across 16 economically diverse countries
Source: BMC Public Health. 2024 Oct 1;24:2678. doi: 10.1186/s12889-024-20147-y (PMC11443786; doi:10.1186/s12889-024-20147-y)
Supplement: Supplementary file 1 — Supplementary Material 1. [file 12889_2024_20147_MOESM1_ESM.docx]

**SUPPLEMENTARY MATERIAL**

**LEGENDS OF TABLES AND FIGURES**

**TABLES**

**S1 Table:** Descriptive statistics of in-person GP challenge type sample for analysis

**S2 Table:** Descriptive statistics of surgical or clinical admission challenge type sample for analysis

**S3 Table:** Descriptive statistics of digital (internet/telephone) appointment challenge type sample for analysis

**S4 Table:** T-test results for sub-sample difference testing

**S5 Table:** Linear regression results of challenge concentration indices on covariates

**S6 Table:** Comprehensive computed concentration index results

**S7 Table:** Variable description for standardization

**S8 Table:** Variable description of external metrics

**S9 Table:** World Bank Income group classification

**FIGURES**

**S1 Figure:** Proportion of respondents challenged by type of care by country

**S2 Figure:** In-person GP challenge inequality by country ranked median income level using restricted standardization

**S3 Figure:** Surgical/clinical challenge inequality by country ranked median income level using restricted standardization

**S4 Figure:** Digital challenge inequality by country ranked median income level using restricted standardization

**S5 Figure:** In-person GP challenge inequality by density of medical practitioners per 10,000 of population

**S6 Figure:** Surgical/clinical challenge inequality by density of medical practitioners per 10,000 of population

**S7 Figure:** Digital challenge inequality by density of medical practitioners per 10,000 of population

**S8 Figure:** In-person GP challenge inequality by density of medical practitioners per 10,000 of population using restricted standardization

**S9 Figure:** Surgical/clinical challenge inequality by density of medical practitioners per 10,000 of population using restricted standardization

**S10 Figure:** Digital challenge inequality by density of medical practitioners per 10,000 of population using restricted standardization

**S1 Table: In-person GP subgroup descriptive statistics sample for analysis**

|  | **Median Age (years)** | **Female (%)** | **Household Size (n)** | **Married (%)** | **Median Ideology^2^** | **Median Educ. level^3^** | **Med. Income (PPP$ '000s)** | **Labour force participation (%)** | **Self-reported health (%)** | **No. Chronic conditions (n)** | **COVID risk (%)** | **Willingness to risk health^4^** | **Sample size (n)** |
| --- | --- | --- | --- | --- | --- | --- | --- | --- | --- | --- | --- | --- | --- |
| Australia | 44(30-59) | 51·2(50) | 2·3(1·4) | 61·8(48) | 5(4-6) | 3(3-4) | 34·9(20-57) | 64·4(47) | 69·7(21) | 0·77(1·0) | 52·3(27) | 3·5(2·5) | 1508 |
| Brazil | 41(30-55) | 49·4(50) | 2·7(1·3) | 61·4(48) | 5(3-8) | 3(2-3) | 8·8(5-14) | 66·7(47) | 72·4(26) | 0·71(0·9) | 38·1(30) | 3·2(3·0) | 905 |
| Canada | 47(32-61) | 64·4(47) | 2·1(1·0) | 49·6(50) | 5(4-7) | 3(3-4) | 35·7(20-70) | 63·5(48) | 68·7(18) | 0·85(0·9) | 42·3(25) | 3·9(2·4) | 962 |
| Chile | 42(30-56) | 52·8(49) | 2·7(1·2) | 52·4(49) | 5(3-5) | 3(2-4) | 12·1(7-24) | 71·4(45) | 72·2(23) | 0·85(0·9) | 43·2(27) | 3·2(2·7) | 1097 |
| China | 44(28-54) | 46·5(49) | 2·8(1·3) | 74·4(43) | ·· | 3(3-3) | 20·5(15-32) | 73·3(44) | 77·4(22) | 0·37(0·6) | 36·2(32) | 3·8(3·1) | 1080 |
| Colombia | 50(31-62) | 48·8(50) | 3·2(3·2) | 54·6(49) | 5(3-6) | 3(2-3) | 7·2(4-12) | 71·9(44) | 56·7(40) | 0·50(0·9) | 38·1(25) | 3·6(3·1) | 1056 |
| France | 49(34-66) | 56·3(49) | 1·9(0·6) | 68·1(46) | 6(5-8) | 3(2-4) | 32·5(20-47) | 55·7(49) | 62·9(26) | 0·72(0·8) | 46·1(21) | 3·9(3·1) | 803 |
| Ghana | 30(27-35) | 18·8(39) | 4·3(2·2) | 52·8(49) | 6(5-7) | 4(3-4) | 1·2(0·5-5) | 76·5(42) | 84·7(15) | 0·18(0·4) | 21·9(27) | 3·4(3·1) | 945 |
| India | 31(25-37) | 41·8(49) | 6·4(8·9) | 50·3(50) | 7(6-9) | 3(2-4) | 4·5(2-13) | 83·1(37) | 55·2(36) | 0·64(1·0) | 55·4(31) | 6(2·8) | 1299 |
| Italy | 52(41-62) | 55(49) | 2·5(1·3) | 64·2(47) | 5(4-7) | 3(3-3) | 24·1(15-42) | 57·4(49) | 68·6(23) | 0·71(0·8) | 45·2(25) | 3·2(2·7) | 883 |
| Japan | 59(43-65) | 38·6(48) | 2·1(1·2) | 66(47) | 5(5-6) | 3(3-4) | 31·1(15-45) | 74·9(43) | 70·3(21) | 0·68(0·8) | 47·6(21) | 4·3(2·4) | 626 |
| South Africa | 36(28-47) | 47·3(49) | 3·1(1·7) | 69·1(46) | 5(4-6) | 3(2-3) | 11·5(0·9-21) | 76·1(42) | 75·1(24) | 0·66(1·4) | 33·2(29) | 2·9(2·5) | 1068 |
| Spain | 48(35-62) | 49·6(50) | 2·4(1·9) | 67·4(46) | 4(2-6) | 3(3-4) | 30·5(22-44) | 66·2(47) | 75·1(22) | 0·68(0·8) | 53(26) | 2·9(2·5) | 999 |
| Uganda | 28(25-34) | 30·2(45) | 3·5(2·4) | 29·9(45) | 5(4-7) | 3(3-3) | 0·2(0·09-0·6) | 92·7(26) | 78·3(19) | 0·59(1·0) | 18·5(20) | 3·4(3·0) | 999 |
| UK | 45(39-64) | 36·1(48) | 1·9(0·9) | 66·4(47) | 5(4-7) | 3(3-4) | 33·2(13-50) | 66·8(47) | 64·3(23) | 0·57(0·9) | 42·7(23) | 4·2(2·5) | 768 |
| US | 41(29-54) | 49·1(50) | 2·4(2·7) | 62·7(48) | 5(4-8) | 4(3-4) | 44·2(18-78) | 76·7(42) | 73·2(23) | 0·92(1·0) | 40·2(29) | 4(2·9) | 1000 |
| \| 1. Figures are presented as mean(SD) and median(IQR) 2. Respondents were asked to rate their political ideology from 1 (left) to 10 (right). Responses were not collected in China. 3. 2 = primary schooling completed; 3 = secondary school completed; 4 = university completed 4. Respondents were asked how willing they were to risk their health from 0 to 10. \| \| --- \| | | | | | | | | | | | | | |

**S2 Table: Surgical and clinical challenges subgroup descriptive statistics sample for analysis**

|  | **Median Age (years)** | **Female (%)** | **Household Size (n)** | **Married (%)** | **Median Ideology^2^** | **Median Educ. level^3^** | **Med. Income (PPP$ '000s)** | **Labour force participation (%)** | **Self-reported health (%)** | **No. Chronic conditions (n)** | **COVID risk (%)** | **Willingness to risk health^4^** | **Sample size (n)** |
| --- | --- | --- | --- | --- | --- | --- | --- | --- | --- | --- | --- | --- | --- |
| Australia | 41(29-55) | 47·3(49) | 2·3(1·7) | 61·8(48) | 5(5-7) | 3(3-4) | 34·9(19-58) | 67·3(46) | 68·4(22) | 0·77(0·9) | 53·3(26) | 3·9(2·7) | 782 |
| Brazil | 41(30-55) | 48·1(49) | 2·7(1·2) | 61·5(48) | 5(4-8) | 3(2-3) | 7·5(4-14) | 68·5(46) | 71·7(27) | 0·70(0·9) | 40(30) | 3·3(3·0) | 699 |
| Canada | 51(32-55) | 56·7(49) | 2(1·0) | 48(50) | 6(5-8) | 3(3-4) | 35·7(12-70) | 58·9(49) | 64·8(21) | 0·62(0·9) | 51·6(25) | 3·8(2·5) | 433 |
| Chile | 45(32-56) | 52·5(49) | 2·8(1·3) | 60·5(48) | 5(2-5) | 3(2-3) | 10(5-17) | 68·9(46) | 69(25) | 0·93(0·9) | 45·6(27) | 3·4(2·7) | 597 |
| China | 43(27-54) | 49·1(50) | 2·8(1·3) | 73·7(44) | ·· | 3(3-3) | 20·5(15-32) | 75·3(43) | 77·1(23) | 0·38(0·6) | 39·4(32) | 4·2(3·1) | 919 |
| Colombia | 49(31-59) | 52·7(49) | 3·2(3·7) | 59·8(49) | 5(4-6) | 3(2-3) | 7·9(5-12) | 81·3(39) | 47·7(40) | 0·53(0·9) | 39·3(26) | 4·1(3·1) | 762 |
| France | 38(30-63) | 53·5(49) | 1·8(0·6) | 64·7(47) | 6(5-8) | 3(2-3) | 33·4(15-47) | 65·6(47) | 56(28) | 0·50(0·7) | 48·5(19) | 5·1(3·1) | 332 |
| Ghana | 30(27-34) | 17·3(37) | 4·3(2·2) | 51·2(50) | 6(5-7) | 4(3-4) | 0·9(0·5-4) | 77(42) | 85·5(15) | 0·19(0·4) | 22·5(28) | 3·8(3·3) | 671 |
| India | 30(24-35) | 46·4(49) | 6·8(9·1) | 45·2(49) | 7(6-9) | 3(2-4) | 3·2(2-10) | 85·9(34) | 51·1(36) | 0·67(1·0) | 56·3(31) | 6·1(2·7) | 1201 |
| Italy | 51(34-61) | 55·7(49) | 2·5(1·5) | 59·7(49) | 6(5-7) | 3(3-3) | 23·2(16-42) | 63·2(48) | 65·1(25) | 0·65(0·9) | 46·5(24) | 3·7(2·9) | 560 |
| Japan | 51(36-61) | 36·7(48) | 2(1·4) | 65·6(47) | 5(5-6) | 3(3-4) | 31(15-44) | 84(36) | 65·3(23) | 0·70(0·9) | 49·6(21) | 4·8(2·2) | 314 |
| South Africa | 36(27-47) | 51·8(50) | 3·3(1·9) | 68·5(46) | 5(2-7) | 3(2-3) | 4·3(0·7-21) | 75·1(43) | 71·1(25) | 0·73(1·6) | 34·3(30) | 3·3(2·5) | 741 |
| Spain | 50(37-62) | 50·4(50) | 2·5(2·5) | 67·9(46) | 5(2-6) | 3(3-4) | 30·5(17-44) | 65·1(47) | 71·9(24) | 0·71(0·9) | 53·3(26) | 3·3(2·7) | 513 |
| Uganda | 28(25-34) | 23·7(42) | 3·8(2·2) | 31·7(46) | 6(4-7) | 3(3-3) | 0·2(0·06-0·4) | 95·2(21) | 75·7(20) | 0·47(0·9) | 15·9(19) | 3·6(2·9) | 692 |
| UK | 45(39-55) | 32·4(46) | 1·9(1·0) | 66·5(47) | 5(4-7) | 3(3-4) | 40·6(19-50) | 79·5(40) | 69(23) | 0·52(0·9) | 42·3(22) | 4·1(2·6) | 529 |
| US | 36(28-47) | 44·6(49) | 2·6(3·3) | 65·8(47) | 6(5-8) | 4(3-4) | 43·7(16-87) | 82·4(38) | 72·7(25) | 0·81(1·0) | 46·2(30) | 4·6(3·0) | 629 |
| 1. Figures are presented as mean(SD) and median(IQR) 2. Respondents were asked to rate their political ideology from 1 (left) to 10 (right). Responses were not collected in China. 3. 2 = primary schooling completed; 3 = secondary school completed; 4 = university completed 4. Respondents were asked how willing they were to risk their health from 0 to 10. | | | | | | | | | | | | | |

**S3 Table: Digital challenges subgroup descriptive statistics sample for analysis**

|  | **Median Age (years)** | **Female (%)** | **Household Size (n)** | **Married (%)** | **Median Ideology^2^** | **Median Educ. level^3^** | **Med. Income (PPP$ '000s)** | **Labour force participation (%)** | **Self-reported health (%)** | **No. Chronic conditions (n)** | **COVID risk (%)** | **Willingness to risk health^4^** | **Sample size (n)** |
| --- | --- | --- | --- | --- | --- | --- | --- | --- | --- | --- | --- | --- | --- |
| Australia | 42(30-57) | 53·2(49) | 2·3(1·5) | 61·3(48) | 5(4-6) | 3(3-4) | 34·9(19-58) | 66·7(47) | 68·5(21) | 0·78(1·0) | 53(27) | 3·6(2·6) | 1254 |
| Brazil | 41(30-55) | 47·3(49) | 2·6(1·2) | 61·1(48) | 5(3-8) | 3(2-3) | 8·8(4-14) | 67·3(46) | 72·2(27) | 0·68(0·9) | 39·4(30) | 3·3(3·0) | 736 |
| Canada | 46(32-61) | 65·2(47) | 2·1(1·0) | 49·6(50) | 5(4-7) | 3(3-4) | 35·7(20-71) | 63·3(48) | 68·5(19) | 0·86(0·9) | 42·6(26) | 3·9(2·4) | 880 |
| Chile | 44(31-56) | 53·2(49) | 2·7(1·2) | 53·6(49) | 5(3-5) | 3(2-4) | 11·7(6-20) | 71·2(45) | 71·5(24) | 0·86(0·9) | 44(27) | 3·3(2·7) | 966 |
| China | 44(27-56) | 48·2(49) | 2·9(1·3) | 72·6(44) | ·· | 3(3-3) | 20·5(15-32) | 71·4(45) | 76·7(23) | 0·38(0·6) | 39·4(32) | 4·1(3·2) | 1007 |
| Colombia | 50(31-62) | 48·4(50) | 3·2(3·3) | 54·2(49) | 5(3-6) | 3(2-3) | 7·2(4-12) | 71·5(45) | 56·4(40) | 0·50(0·9) | 38(25) | 3·6(3·1) | 1036 |
| France | 43(33-63) | 52·2(49) | 1·8(0·6) | 65·3(47) | 6(5-8) | 3(2-4) | 32·5(15-47) | 62(48) | 60·9(27) | 0·57(0·7) | 46·3(21) | 4·6(3·0) | 568 |
| Ghana | 30(27-35) | 17(37) | 4·3(2·2) | 51·9(49) | 6(5-7) | 4(3-4) | 0·9(0·5-4) | 75·8(42) | 85·2(15) | 0·17(0·4) | 22·2(27) | 3·5(3·2) | 809 |
| India | 31(24-36) | 45·7(49) | 6·5(8·9) | 46·9(49) | 7(5-9) | 3(2-4) | 3·5(2-10) | 84(36) | 53·3(36) | 0·65(1·0) | 55(31) | 5·9(2·8) | 1279 |
| Italy | 52(39-62) | 55·2(49) | 2·5(1·4) | 64·1(48) | 5(4-7) | 3(3-3) | 24·1(16-42) | 59·4(49) | 69·1(23) | 0·67(0·8) | 45·6(25) | 3·3(2·7) | 821 |
| Japan | 49(37-61) | 35·2(47) | 1·9(1·4) | 60·9(48) | 5(5-6) | 3(3-4) | 31·1(15-44) | 80·3(39) | 66·2(23) | 0·51(0·7) | 49·7(22) | 4·7(2·3) | 291 |
| South Africa | 36(26-47) | 52·6(49) | 3·3(1·8) | 67·6(46) | 5(3-7) | 3(2-3) | 5·2(0·8-24) | 75·9(42) | 72·4(25) | 0·74(1·6) | 33·4(31) | 3·3(2·6) | 857 |
| Spain | 48(35-61) | 50·3(50) | 2·4(1·9) | 67·3(46) | 4(2-6) | 3(3-4) | 30·5(19-44) | 66·5(47) | 75·1(22) | 0·68(0·8) | 53·2(26) | 2·9(2·5) | 976 |
| Uganda | 26(25-33) | 21·5(41) | 3·7(2·2) | 40·5(49) | 5(4-7) | 3(3-3) | 0·2(0·09-0·4) | 94·5(22) | 73(20) | 0·54(0·8) | 18·8(20) | 3·9(2·9) | 885 |
| UK | 46(39-58) | 36·1(48) | 1·9(0·9) | 66·4(47) | 5(4-7) | 3(3-4) | 39·1(23-50) | 75·8(42) | 69·2(22) | 0·58(0·9) | 42·8(22) | 3·9(2·6) | 770 |
| US | 39(29-52) | 48·6(50) | 2·5(2·9) | 63·4(48) | 6(4-8) | 4(3-4) | 43·7(18-87) | 79·6(40) | 73·1(23) | 0·87(1·0) | 43·2(29) | 4·3(2·9) | 847 |
| 1. Figures are presented as mean(SD) and median(IQR) 2. Respondents were asked to rate their political ideology from 1 (left) to 10 (right). Responses were not collected in China. 3. 2 = primary schooling completed; 3 = secondary school completed; 4 = university completed 4. Respondents were asked how willing they were to risk their health from 0 to 10. | | | | | | | | | | | | | |

**S4 Table:** **T-test results for sub-sample difference testing**

|  | Included in study (1) | Excluded from study (0) | Difference (0) - (1) | T-statistic | Significance level |
| --- | --- | --- | --- | --- | --- |
| Median age (years) | 39 | 42 | 3 | 24.03 | <0.000 |
| Female (%) | 0.496 | 0.514 | 0.018 | 10.51 | <0.000 |
| Educ. Level | 3.265 | 3.185 | -0.080 | -12.17 | <0.000 |
| Income (PPP$ '000s) | 24043.55 | 26506.42 | 2462.87 | 11.58 | <0.000 |
| Labour force participation (%) | 0.704 | 0.685 | -0.019 | -10.36 | <0.000 |
| Self-reported health (%) | 68.472 | 69.197 | 0.725 | 6.40 | <0.000 |
| No. Chronic conditions (n) | 0.610 | 0.609 | -0.001 | -0.33 | 0.7451 |
| Willingness to risk health | 3.520 | 3.477 | -0.043 | -4.15 | <0.000 |

**S5 Table: Linear regression results: Challenge concentration indices on possible explanatory variables**

|  | (1) | (2) | (3) |
| --- | --- | --- | --- |
|  | In person GP challenge concentration index | Surgical/clinical challenge concentration index | Digital challenge concentration index |
|  |  |  |  |
| Median household income | -1·02e-05* | -1·05e-05** | -9·02e-06* |
| SE | (4·67e-06) | (4·04e-06) | (4·45e-06) |
| T statistic | -2·177 | -2·605 | -2·028 |
| p-value | (0·0575) | (0·0285) | (0·0732) |
|  |  |  |  |
| Health expenditure (2019) | 6·68e-06 | -1·20e-05 | 1·42e-05 |
| SE | (1·34e-05) | (1·16e-05) | (1·28e-05) |
| T statistic | 0·499 | -1·036 | 1·115 |
| p-value | (0·630) | (0·327) | (0·294) |
|  |  |  |  |
| COVID-19 containment policy | -0·00102 | -0·00360 | -0·000393 |
| SE | (0·00348) | (0·00301) | (0·00331) |
| T statistic | -0·295 | -1·197 | -0·119 |
| p-value | (0·775) | (0·262) | (0·908) |
|  |  |  |  |
| Medical practitioner density | 0·00704** | 0·00899*** | 0·00309 |
| SE | (0·00299) | (0·00259) | (0·00285) |
| T statistic | 2·354 | 3·474 | 1·084 |
| p-value | (0·0430) | (0·00700) | (0·306) |
|  |  |  |  |
| Internet access | -0·000538 | -0·00267 | 0·000798 |
| SE | (0·00183) | (0·00158) | (0·00174) |
| T statistic | -0·295 | -1·694 | 0·459 |
| p-value | (0·775) | (0·124) | (0·657) |
|  |  |  |  |
| Corruption perceptions index | -0·000368 | 0·000939 | -0·00237 |
| SE | (0·00312) | (0·00269) | (0·00297) |
| T statistic | -0·118 | 0·348 | -0·797 |
| p-value | (0·909) | (0·735) | (0·446) |
|  |  |  |  |
| Constant | 0·158 | 0·347 | 0·189 |
| SE | (0·248) | (0·215) | (0·236) |
| T statistic | 0·638 | 1·619 | 0·800 |
| p-value | (0·539) | (0·140) | (0·444) |
|  |  |  |  |
| Observations | 16 | 16 | 16 |
| R-squared | 0·566 | 0·807 | 0·613 |
| se pval in parentheses |  |  |  |
| *** p<0·01, ** p<0·05, * p<0·1 |  |  |  |

**S6 Table: Comprehensive computed concentration index results**

|  | **Erreygers Index** | | |  | **Wagstaff Index** | | |
| --- | --- | --- | --- | --- | --- | --- | --- |
|  | **In person GP challenge** | **Surgical/clinical challenge** | **Digital challenge** |  | **In person GP challenge** | **Surgical/clinical challenge** | **Digital challenge** |
| Australia | -0·015 | 0·045 | 0·007 |  | -0·016 | 0·045 | 0·01 |
| Brazil | 0·108 | 0·105 | 0·106 |  | 0·108 | 0·115 | 0·108 |
| Canada | -0·113 | -0·295 | -0·065 |  | -0·12 | -0·295 | -0·099 |
| Chile | 0·174 | 0·036 | 0·074 |  | 0·176 | 0·038 | 0·075 |
| China | -0·043 | -0·033 | -0·008 |  | -0·045 | -0·035 | -0·009 |
| Colombia | 0·194 | 0·057 | 0·17 |  | 0·195 | 0·06 | 0·172 |
| France | -0·139 | -0·206 | -0·148 |  | -0·155 | -0·21 | -0·169 |
| Ghana | -0·004 | 0·056 | 0·082 |  | -0·004 | 0·056 | 0·087 |
| India | 0·001 | 0·021 | 0·111 |  | 0·001 | 0·023 | 0·112 |
| Italy | 0·127 | 0·059 | -0·001 |  | 0·13 | 0·06 | -0·001 |
| Japan | -0·026 | -0·06 | -0·155 |  | -0·038 | -0·071 | -0·204 |
| South Africa | 0·098 | 0·024 | 0·12 |  | 0·114 | 0·026 | 0·161 |
| Spain | 0·078 | 0·038 | 0·092 |  | 0·084 | 0·039 | 0·093 |
| Uganda | 0·133 | 0·188 | 0·15 |  | 0·157 | 0·211 | 0·166 |
| UK | 0·087 | 0·025 | 0·114 |  | 0·124 | 0·032 | 0·114 |
| US | 0·024 | -0·212 | 0·049 |  | 0·025 | -0·212 | 0·058 |

**S7 Table: Variable description for standardization**

| **Variable** | **Description** |
| --- | --- |
| Determinants of health  1. Confounding variables 2. Non-confounding control variables | The following variables were used in the needs-based indirect standardization of the health contacts and health issues variables. This process standardizes the contact and challenge variables by health need within each country, enabling further analysis on an income basis without additional confounding factors. The Dahlgren-Whitehead model of the determinants of health is used as a guideline to indicate factors that determine population health.^38^ Given the age of the model, first published in 1991, we sought additional sources to confirm the relevance of the factors at present, including updated perspectives from the original authors and guidance from the WHO. The WHO categorises the determinants of health into three spheres; the social and economic environment, the physical environment, and individual characteristics and behaviours.^39^ We use an expanded set of variables for standardization owing to the nature of the primary analysis, which asks respondents how they perceived challenges in accessing care. The expanded standardization set captures subjectivity in reporting to allow for an unhindered comparison on income. |
| - 1. Age and Age^2^ | Age plays an important role in the provision of, and need for healthcare services – based on how the human body responds to disease and illness.^40^ The population is vulnerable to different diseases at different age groups. For example, the risk of chronic disease increases with age, those over 50 years are more likely to conduct screening checks for diseases, and the prevalence of venereal disease is expected to be highest between the ages of 16-35.^41–44^ The needs of a retired individual are of course likely to be different to those of a university student, encompassing different lifestyles as well as different physical needs. Age is thus an important factor to consider. The age squared term is included to account for non-linearity in the demand placed on the health system. Age is self-reported in the CANDOUR survey, and is measured in years. |
| 1.2 Gender | Gender affects health needs through biological, social and economic determinants of health. Biological sex impacts the health needs of an individual. There are differences in the biological determinants of health by sex; including genetic vulnerability, hormonal and reproductive elements, and physiological characteristics during the life cycle. For example, the likelihood of a biological woman requiring reproductive health care services greatly exceeds that of a biological man. A number of studies show differential nutritional status by gender,^45^ which necessarily impacts development and health status later in life. Economic elements include the economic roles genders take on and how this may affect the health-seeking behaviours of genders.^42^ Gender was self-reported, with options male, female, other, and prefer not to say included as options in the survey. |
| 1.3 Self-reported Health | Self-reported health is used as a measure of general wellbeing, taken from the EQ-5D-5L questionnaire. Health is reported from 0-100, with 100 being perfect health. The EQ-5D-5L question-set was not utilised in full, as many of the countries included in the analysis do not currently have EQ-5D-5L value sets which are required to compute a comprehensive health value from the inputted questions. While an international value set could be used, this may have the effect of artificially smoothing relative health differences across countries and blunting the desired cross-country analysis. |
| 1.4 Chronic conditions | Diseases are generally accepted to be chronic if they remain for over a year and require ongoing treatment.^46^ In some cases, managing chronic diseases may require additional touchpoints with the health system, increasing the health needs of individuals who suffer from chronic illness. The onset of chronic disease is related to several other social determinants of health,^47^ and must therefore be included in the standardization. In the CANDOUR survey, respondents were asked if they had any of the following underlying health conditions; diabetes, hypertension, heart disease, asthma or chronic respiratory issues, allergies, kidney disease, or other chronic illnesses requiring long term care. The number of chronic illnesses selected by the respondent was used as the chronic conditions variable (ranging from 0 to 7). |
| 1.5 Self-reported COVID-19 Risk | Respondents in the CANDOUR survey were asked how likely they were to catch COVID-19 within the next year, and asked to score the likelihood between 0 and 100. We use this variable as an indicator of respondent’s engagement with the health system at large, and their likelihood to seek out medical attention and care. |
| 1.6 Self-reported behavioural health risks | Respondents in the CANDOUR survey were asked how willing they were to take risks with their health, and to rate their willingness from 0-10, with 10 being very willing to take risks. Alongside self-reported COVID-19 risk, we use this variable to determine risk behaviours of individuals relating to health, and their willingness to seek out medical treatment and care. We propose that those more willing to take risks are likely to have a higher barrier for seeking medical care.^48^ |
| 2.1 Education level | Education is one of the compelling social determinants of health, with established links to a number of other social and economic determinants such as social groupings, income, employment opportunities, and healthy ageing itself.^49^ Several studies link higher educational attainment to better health outcomes.^50–52^ Given its links to other determinants of health, it is important to include in the analysis. In the CANDOUR survey, education level is self-reported as the highest degree or level of education completed. The options range from no formal education, nursery to 8^th^ grade, high school, and various degree levels (associate to doctoral), with intermediate steps (i.e., some high school). The values are recoded into four ordinal categories - less than primary completed; completed primary; completed secondary; completed university. |
| 2.2 Chronic medication | Having already included the number of chronic conditions of respondents as a confounding variable of health need, we include dependence on chronic medications as a control variable. In certain settings, prescription fulfilment may require additional contact with the healthcare system, which we wish to control for in this analysis. Respondents were asked if they regularly took medication for any health conditions in the CANDOUR survey. |
| 2.3 Political ideology | Research has shown that conservative societies are less likely to report poor health than left-wing counterparts.^53–55^ Individually, those who identified more with the right politically were also less likely to report poor health.^53^ It is unlikely that it is political ideology itself that determines health outcomes, but more likely that it is a proxy for certain attitudes and latent beliefs which impact health outcomes, or perceived health levels. In the CANDOUR survey, respondents were asked to rate their political ideology from 0 to 10, with 0 being left and 10 being right. In certain countries, this question was excluded from the survey due to the political climate. |
| 2.4 Marital and partnership status | Marital status has been shown to have an impact on health levels in numerous studies.^56^ Results have shown that being unmarried generally leads to greater increases in mortality and disease for men than for women,^57^ though incidence is greater for both sexes than unmarried counterparts. It is proposed that the decreased incidence associated with marriage is caused by the prevention of certain activities which lead to lifestyle diseases.^58^ Further, certain research suggests that those in ill-health have more difficulty finding a partner and marrying; thereby identifying and selecting the married group for good health.^58^ Respondents were asked if they were married, in a civil partnership, or living with their partner – all of which are associated with similar health benefits in the literature.^59^ |
| 2.5 Household size | During the COVID-19 pandemic, household size was shown to be a significant factor in transmission of the infectious disease.^60^ It stands to reason that larger households have an increased number of potential vectors for infectious diseases. However, severity of the disease was higher when living alone or with three or more individuals compared to two person dwellings.^61^ Those living alone were less likely to experience non-severe COVID than those living in two-person households.^61^ Larger households have been associated with lower consumption of medical care,^62^ however this may be due to confounding with socioeconomic status and the substitution of medical care with home health care. Respondents were asked how many adults lived in their household in the CANDOUR survey. The household size variable was truncated at a maximum of 8, resulting in the truncation of 295 households, representing 1.3% of the dataset. |
| 2.6 Labour force participation and Economic activity | Health is positively correlated with labour force participation, one needs to be well to work. Labour force participation has been shown to have a negative effect on male health but a positive effect on female health.^63^ Given the correlation between labour force participation and socioeconomic status, we control for the variable. We also introduce the variable as quantity of available leisure time is likely to influence subjectivity selecting the degree of challenge faced in accessing care. Respondents were asked if they had worked in the week prior to completing the survey. If they had not worked, respondents were asked why. The labour force is defined as those employed or unemployed and of working age who are working or actively seeking work.^64^ Individuals who were in active employment and those who gave the following reasons for not working in the prior week were included in the labour force; ill with COVID-19 symptoms; ill during the past week; recently made redundant; employer closed temporarily; employer closed permanently; could not arrange transport to place of employment. We determined that answering with these options did not make respondents economically inactive, marking them for exclusion from the labour force. Respondents who gave the following reasons for not working in the prior week were marked as economically inactive and excluded from the labour force; did not want to work at present, currently caring for children or the elderly; retired; afraid of the spread of COVID-19; other. The labour force participation variable was dichotomized on the above basis between economically active and economically inactive individuals. |

**S8 Table: Variable description of external metrics**

| **Variable** | **Description** |
| --- | --- |
| External metrics | We submit six possible external variables to be used as correlates with country concentration indices to compare values across countries as a sensitivity analysis. Each of these variables is used as an alternative to median household income, and tested for a monotonic relationship |
| 1. National health expenditure per capita (2019) | As an alternative to median household income, we offer national per capita health spending, available as one of the World Bank’s World Development indicators.^65^ The most recent year available for all included countries is 2019. Previous research has found that health expenditure are linked to inequality and socioeconomic development,^66^ validating its inclusion as an alternative explanatory variable for testing in a sensitivity analysis. |
| 2. Government COVID response | Government responses to the COVID-19 pandemic included a range of measures to combat the spread of COVID-19. Oxford’s Blavatnik School of Government developed a COVID-19 Government Response tracker.^67^ The dataset builds four indices encompassing various government response measures and rates the government response across the measures, producing a single index. The produced indices are a containment and health index; an economic support index; a stringency index; and an overall government support index. For the purposes of the sensitivity analysis, we use the mean containment index, which details 14 response measures. The closure and containment measures included are school closure policy; workplace closure policy; public event policy; gathering restrictions policy; public transport policy; stay at home policy; internal movement restrictions; and international travel controls. The health policy measures included are the presence of public information campaigns; access to testing and testing policy; policy on contact tracing; facial covering policy; vaccination policy; and policies on the protection of the elderly. Using this combined index as a measure of Government’s COVID-19 response through a health and containment lens, we feel it acts as a viable possibility of an alternative measure which could explain observed inequality between countries in the sensitivity analysis. |
| 3. Median age | At the onset of the COVID-19 pandemic, advanced age was cited as a cause of severe COVID-19.^68^ Using the CIA World Factbook data,^69^ we propose national median age as an alternative explanatory variable that may be linked to systematic inequality in our sensitivity analysis. Median age is taken simply as the median age within each specified country, with the last reported year 2021. |
| 4. Medical practitioner density | The supply of healthcare professionals is necessarily a factor in the availability of appointments, and thus potentially related to the ease with which individuals can access health systems and consume healthcare.^70^ We propose the density of medical practitioners as a potential alternative correlate for inequality in access to care. The variable is taken as the total number of medical practitioners per 10 000 people in a country. Data availability limited the disaggregation of medical practitioners into generalists and specialists. The most recent available data for the selected countries were between 2019 and 2021, accessed via the WHO’s National Health Workforce Accounts data portal.^71^ To reach a common year, 2020 was taken. Countries missing 2020 data, but with 2019 and 2021 data were averaged across the two years. |
| 5. Internet access | Specifically in the digital health channel, however perhaps more broadly, access to the internet is known to be an important marker in accessing care.^72^ To measure internet access, we use the International Telecommunications Union’s digital dashboard.^73^ We use the % of individuals with access to internet within a country as a metric for overall internet access. |
| 6. Corruption index | Corruption plays a larger role in healthcare provision than many would like to admit.^74^ Globally, healthcare sectors have been identified as having high levels of corruption,^75^ while 33% of OECD citizens and 45% of citizens worldwide characterise the healthcare sector as corrupt.^76^ It is possible that corruption in the health sector impedes access to care, and as such, we include it as a possible alternative measure. We use Transparency International’s Corruption Perception Index.^77^ The index combines data from several sources to build out the index, which measures elements of bribery, diversion of public funds, state capture, integrity, red tape and bureaucratic burdens, nepotism, criminal prosecution of corrupt officials, use of public office for private gain, financial disclosure laws, protections for whistle-blowers, and access to information. |

**S9 Table: World Bank Income group classification**

| Income Group | Countries |
| --- | --- |
| Low income | Uganda |
| Lower middle-income | Ghana; India |
| Upper middle-income | Brazil; China; Colombia; South Africa |
| High income | Australia; Canada; Chile; France; Italy; Japan; Spain; United Kingdom of Great Britain; United States of America |

**S1 Figure: Proportion of respondents challenged by type of care by country**

**
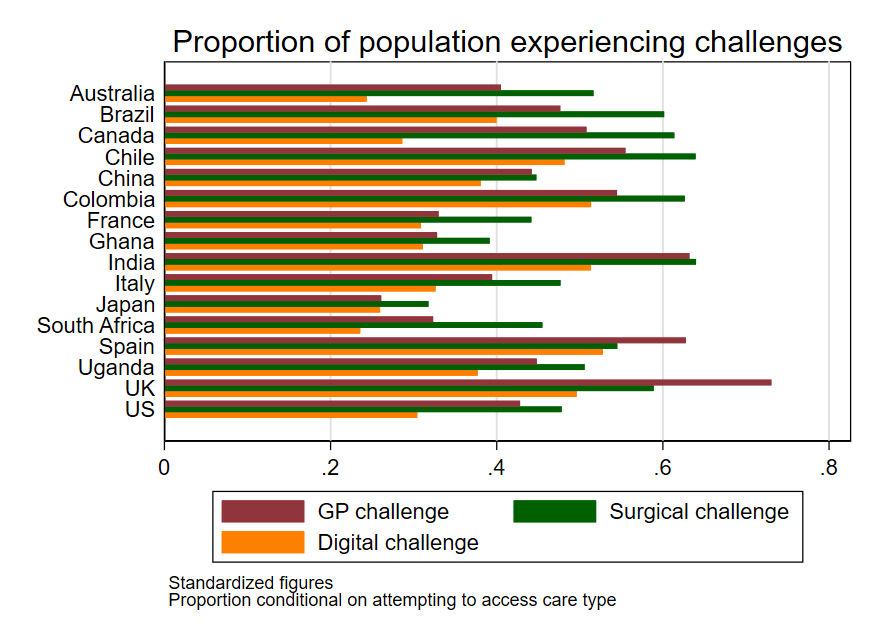
**

**S2 Figure: In-person GP challenge inequality by country ranked median income level using restricted standardization**

**
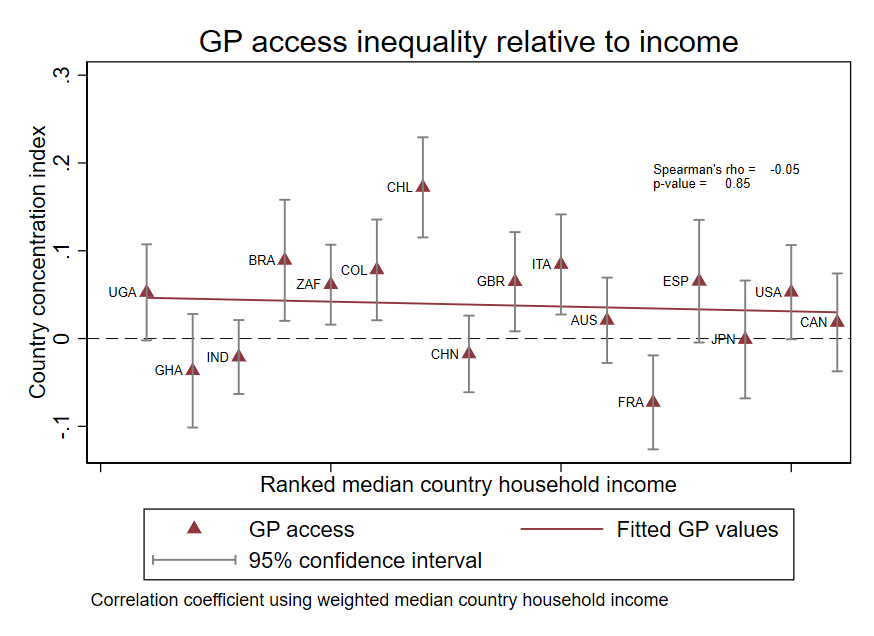
**

**S3 Figure: Surgical/clinical challenge inequality by country ranked median income level using restricted standardization**

**
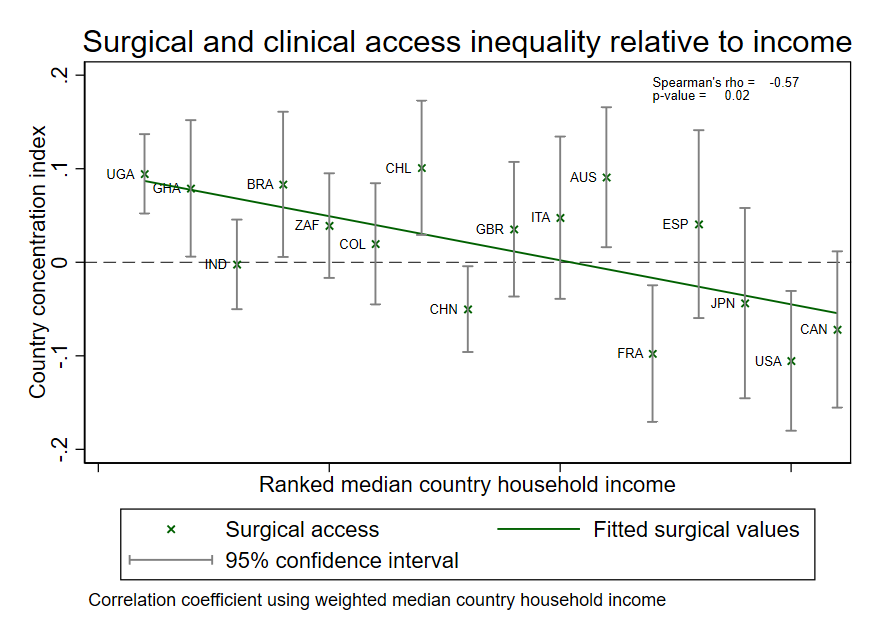
**

**S4 Figure: Digital challenge inequality by country ranked median income level using restricted standardization**

**
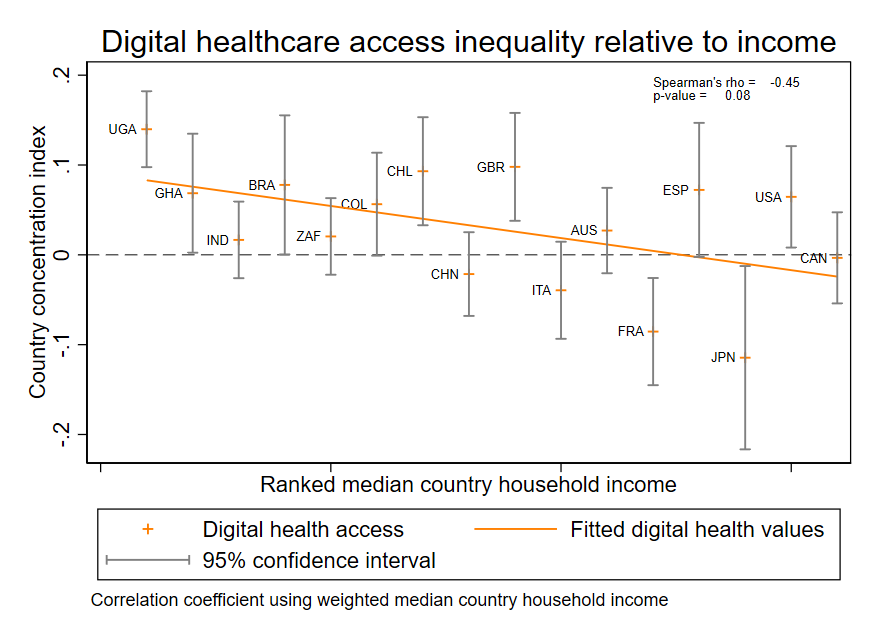
**

**S5 Figure:** **In-person GP challenge inequality by density of medical practitioners per 10,000 of population**

**
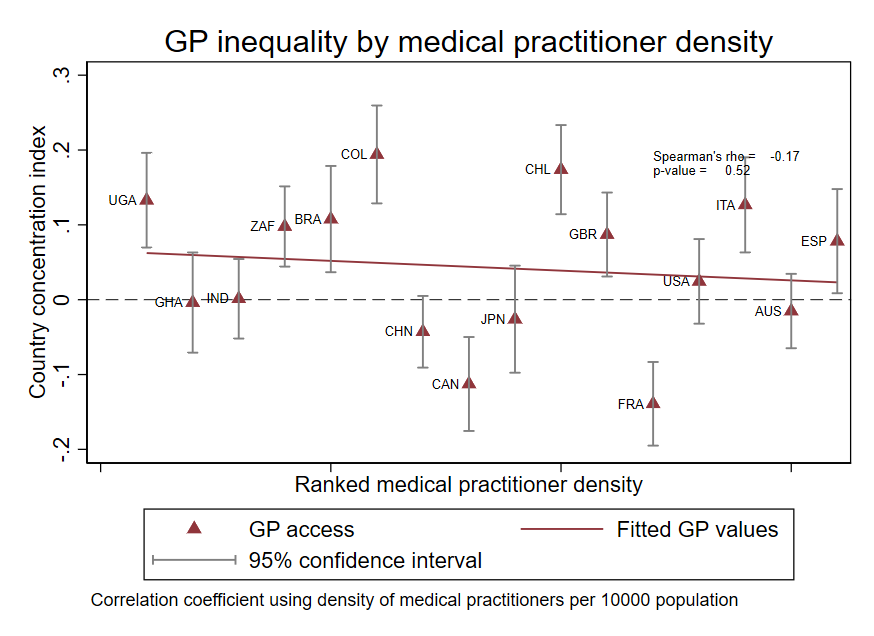
**

**S6 Figure: Surgical/clinical challenge inequality by density of medical practitioners per 10,000 of population**

**
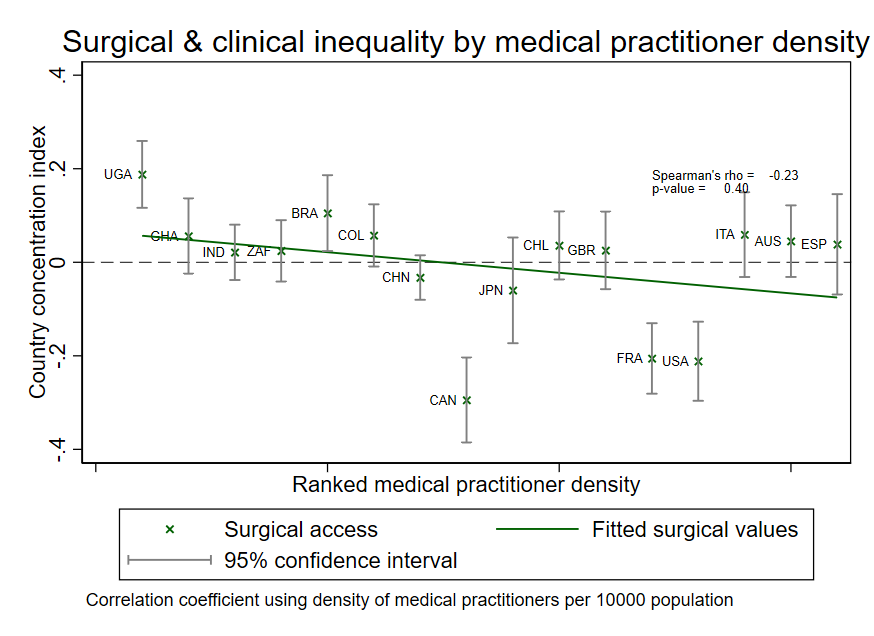
**

**S7 Figure: Digital challenge inequality by density of medical practitioners per 10,000 of population**

**
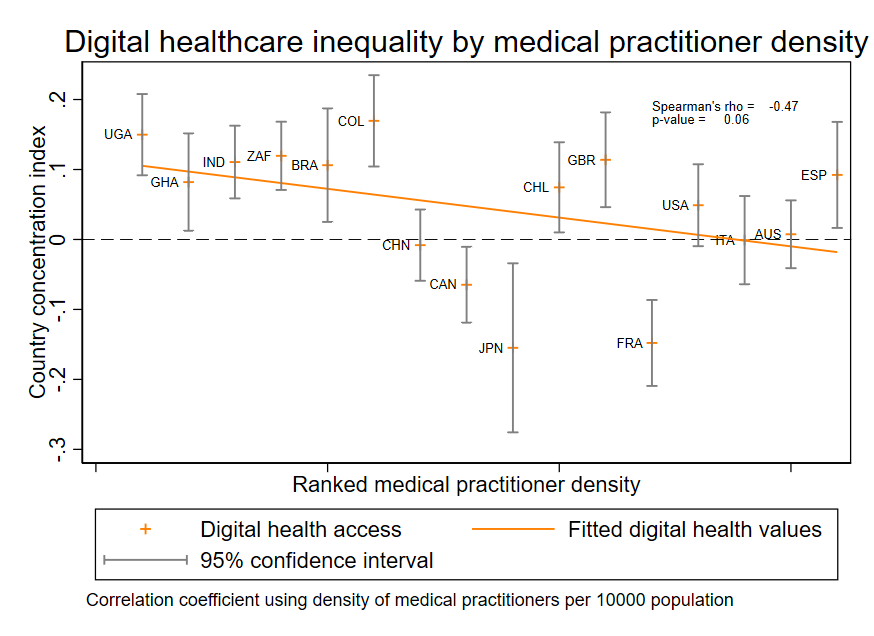
**

**S8 Figure:** **In-person GP challenge inequality by density of medical practitioners per 10,000 of population using restricted standardization**

**
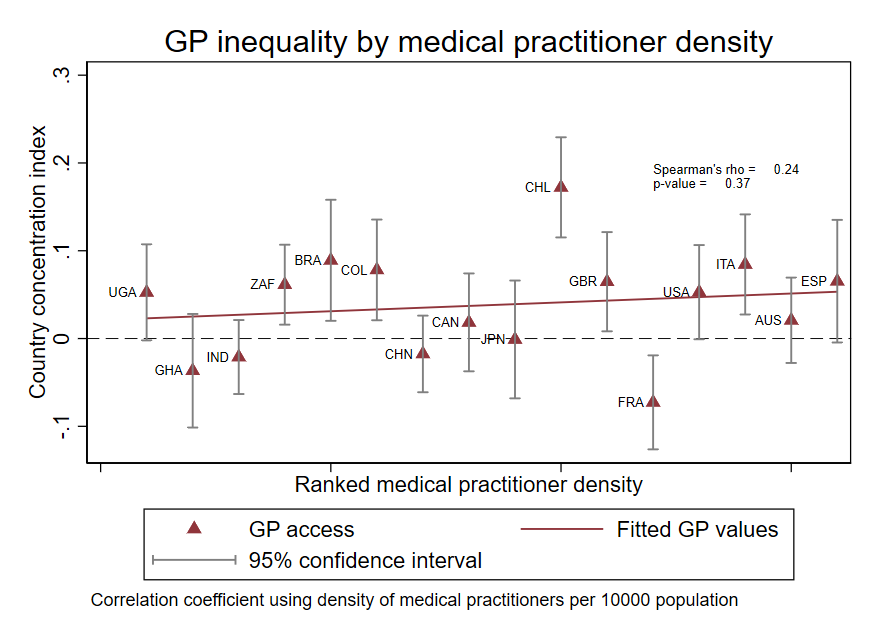
**

**S9 Figure: Surgical/clinical challenge inequality by density of medical practitioners per 10,000 of population using restricted standardization**

**
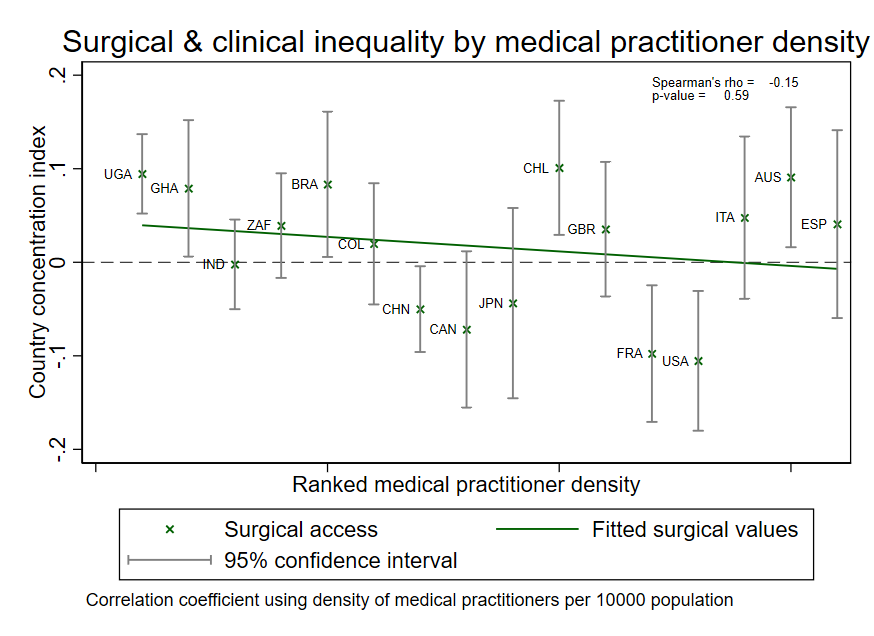
**

**S10 Figure: Digital challenge inequality by density of medical practitioners per 10,000 of population using restricted standardization**

**
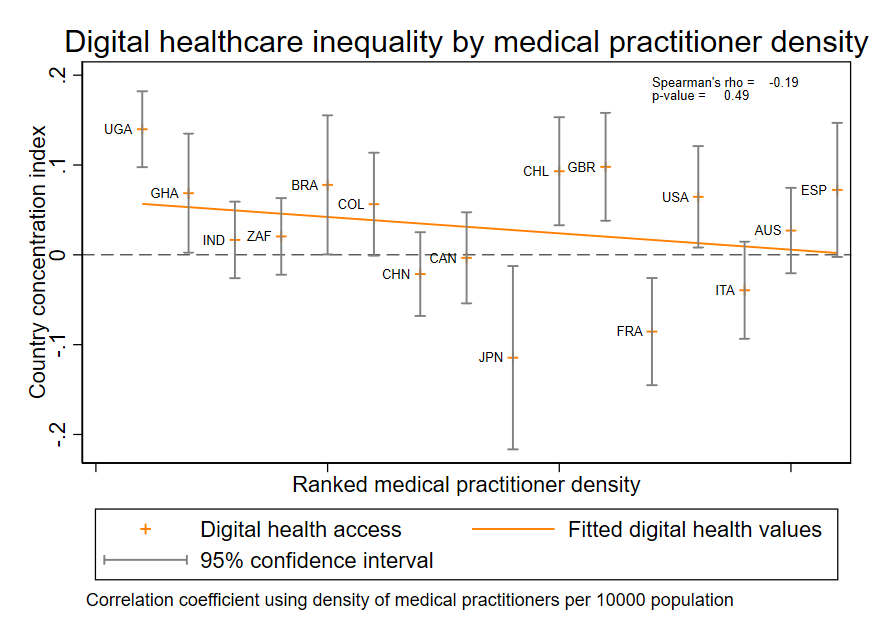
**

**Supplementary references**

38. Dahlgren G, Whitehead M. The Dahlgren-Whitehead model of health determinants: 30 years on and still chasing rainbows. *Public Health*. 2021;199:20-24. doi:10.1016/j.puhe.2021.08.009

39. Committee on Educating Health Professionals to Address the Social Determinants of Health, Board on Global Health, Institute of Medicine, National Academies of Sciences, Engineering, and Medicine. *A Framework for Educating Health Professionals to Address the Social Determinants of Health*. National Academies Press (US); 2016. Accessed November 18, 2023. http://www.ncbi.nlm.nih.gov/books/NBK395983/

40. Geifman N, Cohen R, Rubin E. Redefining meaningful age groups in the context of disease. *Age (Dordr)*. 2013;35(6):2357-2366. doi:10.1007/s11357-013-9510-6

41. Does the Association Between Age and Major Illness Vary by Healthcare System Quality? - Matthew A. Andersson, Lindsay R. Wilkinson, Markus H. Schafer, 2019. Accessed November 18, 2023. https://journals.sagepub.com/doi/10.1177/0164027519864720

42. Deeks A, Lombard C, Michelmore J, Teede H. The effects of gender and age on health related behaviors. *BMC Public Health*. 2009;9(1):213. doi:10.1186/1471-2458-9-213

43. Syrjänen K, Väyrynen M, Castrén O, et al. Sexual behaviour of women with human papillomavirus (HPV) lesions of the uterine cervix. *Br J Vener Dis*. 1984;60(4):243-248. doi:10.1136/sti.60.4.243

44. Weinstock H, Berman S, Cates W. Sexually transmitted diseases among American youth: incidence and prevalence estimates, 2000. *Perspect Sex Reprod Health*. 2004;36(1):6-10. doi:10.1363/psrh.36.6.04

45. Backstrand JR, Allen LH, Pelto GH, Chávez A. Examining the gender gap in nutrition: an example from rural Mexico. *Soc Sci Med*. 1997;44(11):1751-1759. doi:10.1016/s0277-9536(96)00376-0

46. Fernandez-Lazaro CI, García-González JM, Adams DP, et al. Adherence to treatment and related factors among patients with chronic conditions in primary care: a cross-sectional study. *BMC Family Practice*. 2019;20(1):132. doi:10.1186/s12875-019-1019-3

47. Vennu V, Abdulrahman TA, Alenazi AM, Bindawas SM. Associations between social determinants and the presence of chronic diseases: data from the osteoarthritis Initiative. *BMC Public Health*. 2020;20(1):1323. doi:10.1186/s12889-020-09451-5

48. Taber JM, Leyva B, Persoskie A. Why do People Avoid Medical Care? A Qualitative Study Using National Data. *J Gen Intern Med*. 2015;30(3):290-297. doi:10.1007/s11606-014-3089-1

49. Health TLP. Education: a neglected social determinant of health. *The Lancet Public Health*. 2020;5(7):e361. doi:10.1016/S2468-2667(20)30144-4

50. Raghupathi V, Raghupathi W. The influence of education on health: an empirical assessment of OECD countries for the period 1995–2015. *Archives of Public Health*. 2020;78(1):20. doi:10.1186/s13690-020-00402-5

51. Wu YT, Daskalopoulou C, Muniz Terrera G, et al. Education and wealth inequalities in healthy ageing in eight harmonised cohorts in the ATHLOS consortium: a population-based study. *Lancet Public Health*. 2020;5(7):e386-e394. doi:10.1016/S2468-2667(20)30077-3

52. Gumà J, Solé-Auró A, Arpino B. Examining social determinants of health: the role of education, household arrangements and country groups by gender. *BMC Public Health*. 2019;19(1):699. doi:10.1186/s12889-019-7054-0

53. Subramanian SV, Huijts T, Perkins JM. Association between political ideology and health in Europe. *Eur J Public Health*. 2009;19(5):455-457. doi:10.1093/eurpub/ckp077

54. Smith GD, Dorling D. “I’m all right, John”: voting patterns and mortality in England and Wales, 1981-92. *BMJ*. 1996;313(7072):1573-1577. doi:10.1136/bmj.313.7072.1573

55. Dorling D, Smith GD, Shaw M. Analysis of trends in premature mortality by Labour voting in the 1997 general election. *BMJ*. 2001;322(7298):1336-1337. doi:10.1136/bmj.322.7298.1336

56. Robards J, Evandrou M, Falkingham J, Vlachantoni A. Marital status, health and mortality. *Maturitas*. 2012;73(4):295-299. doi:10.1016/j.maturitas.2012.08.007

57. Wang Y, Jiao Y, Nie J, et al. Sex differences in the association between marital status and the risk of cardiovascular, cancer, and all-cause mortality: a systematic review and meta-analysis of 7,881,040 individuals. *Global Health Research and Policy*. 2020;5(1):4. doi:10.1186/s41256-020-00133-8

58. Verbrugge LM. Marital Status and Health. *Journal of Marriage and Family*. 1979;41(2):267-285. doi:10.2307/351696

59. Kim A, Lee JA, Park HS. Health behaviors and illness according to marital status in middle-aged Koreans. *Journal of Public Health*. 2018;40(2):e99-e106. doi:10.1093/pubmed/fdx071

60. Liu P, McQuarrie L, Song Y, Colijn C. Modelling the impact of household size distribution on the transmission dynamics of COVID-19. *Journal of The Royal Society Interface*. 2021;18(177):20210036. doi:10.1098/rsif.2021.0036

61. Gillies CL, Rowlands AV, Razieh C, et al. Association between household size and COVID-19: A UK Biobank observational study. *J R Soc Med*. 2022;115(4):138-144. doi:10.1177/01410768211073923

62. Halliday TJ, Park M. Household Size, Home Health Care, and Medical Expenditures. *Working Papers*. Published online November 3, 2009. Accessed November 18, 2023. https://ideas.repec.org//p/hai/wpaper/200916.html

63. Cai L. The relationship between health and labour force participation: Evidence from a panel data simultaneous equation model. *Labour Economics*. 2010;17(1):77-90. doi:10.1016/j.labeco.2009.04.001

64. Sangha H, Riegler R. Can globalisation promote female labour force participation? *International Journal of Development Issues*. 2020;19(3):303-322. doi:10.1108/IJDI-07-2019-0135

65. World Development Indicators DataBank. 2018. Accessed 1 March 2023 http://databank.worldbank.org/data/reports.aspx?source=world-development-indicators.

66. Peña-Sánchez AR, Ruiz-Chico J, Jiménez-García M. Dynamics of Public Spending on Health and Socio-Economic Development in the European Union: An Analysis from the Perspective of the Sustainable Development Goals. *Healthcare (Basel)*. 2021;9(3):353. doi:10.3390/healthcare9030353

67. Hale T, Angrist N, Goldszmidt R, et al. A global panel database of pandemic policies (Oxford COVID-19 Government Response Tracker). *Nat Hum Behav*. 2021;5(4):529-538. doi:10.1038/s41562-021-01079-8

68. Starke KR, Reissig D, Petereit-Haack G, Schmauder S, Nienhaus A, Seidler A. The isolated effect of age on the risk of COVID-19 severe outcomes: a systematic review with meta-analysis. *BMJ Global Health*. 2021;6(12):e006434. doi:10.1136/bmjgh-2021-006434

69. CIA. The World Factbook 2021. Washington, DC: Central Intelligence Agency, 2021. The World Factbook 2021. Washington, DC: Central Intelligence Agency, 2021.

70. Léonard C, Stordeur S, Roberfroid D. Association between physician density and health care consumption: A systematic review of the evidence. *Health Policy*. 2009;91(2):121-134. doi:10.1016/j.healthpol.2008.11.013

71. World Health Organisation. Time series: From year 2000. Global Health Workforce Statistics in Global Health Observatory data repository: https://apps.who.int/gho/data/node.main.HWFGRP?lang=en NHWA data portal: <https://apps.who.int/nhwaportal/> Accessed 27 Sep 2023

72. Eruchalu CN, Pichardo MS, Bharadwaj M, et al. The Expanding Digital Divide: Digital Health Access Inequities during the COVID-19 Pandemic in New York City. *J Urban Health*. 2021;98(2):183-186. doi:10.1007/s11524-020-00508-9

73. ITU. Digital Development Dashboard. 2023. https://www.itu.int/en/ITU-D/Statistics/Dashboards/Pages/Digital-Development.aspx

74. National Academies of Sciences E, Division H and M, Services B on HC, Health B on G, Globally C on I the Q of HC. The Critical Health Impacts of Corruption. In: *Crossing the Global Quality Chasm: Improving Health Care Worldwide*. National Academies Press (US); 2018. Accessed November 18, 2023. https://www.ncbi.nlm.nih.gov/books/NBK535646/

75. Transparency International. Global Corruption Report 2006: Corruption and health - Publications. Transparency.org. Published February 1, 2006. Accessed November 18, 2023. https://www.transparency.org/en/publications/global-corruption-report-2006-corruption-and-health

76. OECD (Organisation for Economic Co-operation and Development). Tackling wasteful spending on health, highlights. Paris, France: OECD; 2017.

77. Transparency International. Corruptions perception index (2021) by Transparency International is licensed under CC BY 4.0
